# To: Memory box: possibilities to support grief in the intensive care unit during the COVID-19 pandemic

**DOI:** 10.5935/0103-507X.20210044

**Published:** 2021

**Authors:** Sora Yasri, Viroj Wiwanitkit

**Affiliations:** 1 Primary Consultant Center - Bangkok, Thailand.; 2 Dr DY Patil University - Pune, India.

**Dear editor,**

We would like to share ideas on the publication “Memory box: possibilities to support grief in the intensive care unit during the COVID-19 pandemic.”^(1)^ Luiz et al. reported on the use of Memory box as a tool to support grief, and concluded that “Providing bereavement support is one of the possibilities we consider when fighting this pandemic perspective”.^(1)^ Requirement of intensive care and death after COVID-19 illness can result in grief among patients’ cousins and friends.^(2)^ There is no doubt that this may provide a tool for mental support. Nevertheless, there are some concerns. First, the sanitation management of the Memory box is a very important issue, as contamination might occur and become a source of disease spreading. Second, the collections in the box might be useful but might lead to an awful picture if there are numerous stories there. It is also controversial if it would not result in a violation of the patients’ and the dead person’s privacy.
